# Exercise instruction during haemodialysis treatment after changes to the insurance regime: a nationwide questionnaire survey in Japan

**DOI:** 10.1038/s41598-024-59995-4

**Published:** 2024-04-22

**Authors:** Tadashi Sofue, Ryota Matsuzawa, Hiroki Nishiwaki, Yohei Tsuchida, Keisei Kosaki, Junichi Hoshino, Ichiei Narita, Kunihiro Yamagata

**Affiliations:** 1 Department of Cardiorenal and Cerebrovascular Medicine, Kagawa University, 1750-1 Ikenobe, Miki-Chou, Kida-Gun, Kagawa 761-0793 Japan; 2 Department of Physical Therapy, School of Rehabilitation, Hyogo Medical University, Kobe, Japan; 3 Division of Nephrology, Department of Internal Medicine, Showa University Fujigaoka Hospital, Yokohama, Japan; 4 Department of Nephrology, Shinrakuen Hospital, Niigata, Japan; 5 Institute of Health and Sport Sciences, University of Tsukuba, Tsukuba, Ibaraki Japan; 6 Department of Nephrology, Tokyo Women’s Medical University, Tokyo, Japan; 7 Division of Clinical Nephrology and Rheumatology, Niigata University Graduate School of Medical and Dental Sciences, Niigata, Japan; 8 Department of Nephrology, Faculty of Medicine, University of Tsukuba, Tsukuba, Japan

**Keywords:** Exercise instruction, Haemodialysis, Insurance claim, Questionnaire survey, Kidney diseases, Renal replacement therapy

## Abstract

In April 2022, an additional medical fee for exercise instruction during haemodialysis treatment was approved for insurance claims in Japan. We conducted a questionnaire survey to investigate the current situation regarding exercise therapy during haemodialysis treatment after this change. Questionnaires were mailed to 4257 haemodialysis facilities, almost all the haemodialysis facilities in Japan, on January 31, 2023. In total, 1657 facilities responded, of which 550 (33%) provided exercise instruction during haemodialysis treatment, and 65% of these claimed the new fee. Of the 550 facilities that had claimed the fee at the time of survey, 245 (55%) started exercise instruction in April 2022 or later. Exercise instruction focused on resistance training (81%) and aerobic exercise (62%) for 20–30 min (66%) three times a week (80%). The instructors included physicians in 45% of facilities, nurses in 74%, and physical therapists in 36%. Efficacy was evaluated in 76% of the facilities providing instruction, mainly by assessing change in muscle strength (49%). Overall, 39% of facilities had experienced some adverse events, but none were life-threatening. In conclusion, after the change in the insurance regime, exercise instruction during haemodialysis treatment has become more popular, and more patients on haemodialysis are undergoing exercise therapy.

## Introduction

The prevalence of sarcopenia in patients on haemodialysis is high, ranging from 25.6 to 34.6%^1,2^. Frailty and sarcopenia in patients on haemodialysis are risks for poor survival outcomes^3^. Exercise therapy for patients on haemodialysis has been reported to be effective in improving survival prognosis, physical quality of life, and levels of physical activity^4–6^. In 2018, the Japanese Society of Renal Rehabilitation (JSRR) published clinical practice guidelines for renal rehabilitation^5^. This recommends exercise therapy for patients on haemodialysis and provides specific examples of exercise regimens, including aerobic exercise and resistance training.

In April 2022, an additional medical fee for exercise instruction during haemodialysis treatment was approved for insurance claims in Japan^7,8^. This additional fee can be claimed when physicians, physical therapists (PTs), occupational therapists (OTs), or nurses who have received specific training in renal rehabilitation from the JSRR provide training to patients on haemodialysis during their treatment. This training is not limited to exercise therapy and can also include nutritional instruction. Exercise therapy could be performed at times other than during haemodialysis. Claims can be made when exercise instruction is provided for at least 20 consecutive minutes during one haemodialysis treatment and can be made for no more than 90 days.

Exercise therapy during haemodialysis treatment in Japan is expected to have been popularized by the ability to claim fees for exercise instruction during haemodialysis treatment. The implementation rate of exercise therapy during haemodialysis in each region has been reported^9,10^. However, it is unclear what this will mean in practice, especially the details of the exercise therapy, and the occurrence of adverse events. We therefore conducted a descriptive study using a nationwide questionnaire survey among almost all haemodialysis facilities in Japan to investigate the current provision of exercise therapy during haemodialysis treatment since the change in ability to claim.

## Materials and methods

### Study design and setting

This nationwide questionnaire-based cross-sectional survey was conducted in Japan by the survey research working group of the academic committee of the JSRR. The survey period was from January 31 to March 31, 2023.

Questionnaires were sent to almost all 4257 haemodialysis facilities in Japan that were members of the Japanese Society for Dialysis Therapy. There was no financial incentive for participation in this survey. To examine whether the responding facilities were representative of the surveyed facilities, we extracted facility data for 2021 from a newly developed web-based system owned by the Japanese Society for Dialysis Therapy: the Web-based Analysis of Dialysis Data Archives system, Ver. 2.1^11^.

### Questionnaire

Members of the survey research working group of the JSRR developed the questionnaire and conducted the survey. The web- or paper-based questionnaire consisted of 27 items (all items about exercise instruction, 17 about patients on haemodialysis, three about patients with non-dialysis chronic kidney disease (CKD), and seven about the facility itself). The content of the questionnaire is shown in Table 1. Questionnaires were mailed on the first day of the survey period, and responses were made either by returning the questionnaires or via the web, with IDs assigned to responding facilities to avoid duplicate responses.

**Table 1:** Items included in the questionnaire.

*Your willingness to return the completed questionnaire indicates your consent to participate in this study
[Items 1 to 17 are questions about patients with maintenance haemodialysis]
Item 1 Does your facility provide exercise instruction during haemodialysis treatment?
Yes/No
If you answered 'yes' to Q1, please answer Q2–Q15; if you answered 'no', please answer Q16–Q17
Item 2 Does your facility claim the medical treatment fee for “exercise instruction during haemodialysis treatment?”
Yes/No
Item 3 Which professions provide exercise instruction during haemodialysis treatment in your facility? (Multiple answers possible)
Physician
Nurse, nursing assistant
Clinical engineer
Physical therapist
Occupational therapist
Health fitness programmer
Pharmacist
Nutritionist
Others
Item 4 For how many patients on haemodialysis does your facility provide exercise instruction during haemodialysis treatment as of 31/12/2022? (Please indicate the number)
()
Item 5 When did your facility start providing exercise instruction during haemodialysis treatment?
Before March 2022 (before changes to the claims regime)
In or after April 2022 (after changes to the claims regime)
Item 6 When does your facility provide exercise instruction to outpatients on haemodialysis? (Multiple answers allowed)
During haemodialysis treatment
Before/after haemodialysis treatment on haemodialysis days
Non-dialysis days
Exercise instruction is not provided
Item 7 If your facility performs an evaluation of effectiveness before and after the start of exercise instruction, please indicate which items are used to determine effectiveness. (Multiple answers allowed)
Exercise tolerance (CPX; cardiopulmonary exercise test)
Exercise tolerance (e.g. 6-min walk test or incremental shuttle walking test)
Short Physical Performance Battery (SPPB)
Evaluation of muscle strength
Evaluation of nutrition status
Evaluation of ADL/QOL
Item 8 Which professions evaluate the effectiveness of exercise instruction? (Multiple answers allowed)
No evaluation
Physician
Nurse, nursing assistant
Clinical engineer
Physical therapist
Occupational therapist
Health fitness programmer
Pharmacist
Nutritionist
Others
Item 9 Please indicate the type of exercise instruction provided during haemodialysis treatment. (Multiple answers allowed)
Aerobic exercise
Upper limb resistance training
Lower limb resistance training
Balance exercise
Group exercise
Nutrition instruction
Others
Item 10 Please indicate the frequency of exercise instruction during haemodialysis treatment
Less than once a week
Once a week
Twice a week
Three times a week
Item 11 Please indicate the approximate duration of exercise instruction during haemodialysis treatment
Less than 20 min
20–30 min
30–40 min
40–60 min
Over 60 min
Item 12 Do you continue to perform exercise instruction during haemodialysis treatment after the 90-day claimable period?
Yes/No/Undecided
Item 13 Do you feel that exercise instruction during haemodialysis is useful for patients on haemodialysis?
Very effective
Effective
Undetermined
Not effective
Not very effective
Item 14 If you have experienced any adverse events during provision of exercise therapy during haemodialysis treatment, please indicate the specific details. (Multiple answers allowed)
Never experienced
Blood pressure decreased
Blood pressure increased
Abnormal heart rate/arrhythmia
Appearance of trouble in circulatory system of haemodialysis
Muscle/joint pain
Falls/trauma
Discomfort/dizziness
Skin problems
Development of angina/myocardial infarction
Development of cerebrovascular disease
Others (please specify)
Item 15 Do you follow the clinical practice guidelines for renal rehabilitation in providing exercise instruction during haemodialysis treatment?
Yes/No/Not sure
Only those who answered “No” in Item 1 responded to Items 16, 17
Item 16 Please indicate the reasons for not providing exercise instruction during haemodialysis treatment
No specific reason
Not interested
No patients wanting exercise therapy
I do not know how to do it
No equipment
Unable to satisfy the claim criteria
Short-staffed
Others
Item 17 Do you plan to start providing exercise instruction during haemodialysis treatment in the future?
Yes/no/undecided
[Items 18 to 20 are questions about non-dialysis CKD patient]
Item 18 Does your facility provide exercise therapy for non-dialysis CKD patients?
-Yes/No
Item 19 If you answered yes to Item 18, for how many non-dialysis CKD patients does your facility provide exercise therapy? (Please indicate the number)
()
Item 20 If you answered no to Item 18, please indicate the reasons for not providing exercise therapy
No specific reason
Not interested
No patients wanting exercise therapy
I do not know how to do it
No equipment
Because the additional fee cannot be claimed for non-dialysis CKD patients
Short-staffed
Others
[Items 21 to 25 are questions about your facility]
Item 21 Please indicate the type of facility
University hospital
Other hospital
Clinic
Other
Item 22 How many inpatient beds does your facility have? (Please indicate the number)
()
Item 23 How many haemodialysis beds does your facility have? (Please indicate the number)
()
Item 24 How many maintenance haemodialysis patients does your facility treat? (Please indicate the number)
()
Item 25 Please give your facility’s name
()
Item 26 Please give the name of the respondent
()
Item 27 Please give the respondent’s e-mail
()

### Ethical issues

This study was conducted in accordance with the Declaration of Helsinki (revised Fortaleza 2013) and the “Ethical guidelines for medical and biological research involving human subjects”^12^. Informed consent was obtained from all study participants. In the primary survey, consent to participate in this study was obtained by returning the completed questionnaire. A secondary survey was conducted to find out more about the 18 moderate-to-severe adverse events reported by 18 facilities in the primary survey. The secondary survey included patient information and was therefore ethically approved by the Kagawa University Ethics Committee [Reference number: 2023-132]. Patients included in the secondary study were guaranteed the opportunity to be excluded from the study using an opt-out consent approach.

### Claims requirements

The Japanese Ministry of Health, Labour and Welfare approved insurance claims for exercise instruction during haemodialysis treatment in April 2022^7,8^. The details of claims requirements are shown in Supplementary Table S1.

### Statistical analysis

Normally distributed variables are expressed as the mean and standard deviation and non-normally distributed variables as the median and interquartile range (IQR). Categorical variables were compared between groups using the chi-squared test and Fisher’s exact test. Continuous variables were compared between groups using the Mann–Whitney test. All analyses used SPSS for Windows software (version 26.0; IBM Japan, Tokyo, Japan). A p-value of < 0.05 was considered statistically significant.

## Results

### Characteristics of facilities

Of the 4257 facilities surveyed, 1657 (39%) responded. The characteristics of the surveyed and responding facilities are shown in Table 2. There were no apparent differences between surveyed and responding facilities in terms of type of facility and patients on haemodialysis but the number of haemodialysis beds was larger in responded facilities than in surveyed facilities. Almost half of the responding facilities were hospitals. A total of 550 facilities, including 266 hospitals, 281 clinics, and three facilities with no given facility type, responded that they provide exercise instruction during haemodialysis treatment. This means that 33% of responding facilities and 13% of surveyed facilities provided this instruction. Facilities providing exercise instruction had fewer inpatient beds, more haemodialysis beds and more patients on haemodialysis than those not providing exercise instruction (Supplementary Table S2).

**Table 2:** Characteristics of surveyed and responding facilities.

	Surveyed facilities* (n = 4455)	Responding facilities (n = 1657)	P value
Type of facility			
Clinic	2133 (48%)	797 (48%)	0.55
Hospital	2322 (52%)	838 (51%)	
Number of haemodialysis beds			
< 24	1862 (42%)	600 (36%)	< 0.01
25–49	1861 (42%)	681 (41%)	
50–99	642 (14%)	278 (17%)	
> 100	88 (2%)	64 (4%)	
Number of haemodialysis beds, average (SD)	32 (23)	36 (27)	< 0.01
Number of patients on haemodialysis			
< 50	1671 (37%)	569 (34%)	0.16
51–100	1593 (36%)	574 (35%)	
> 101	1190 (27%)	466 (28%)	
Number of patients on haemodialysis, average (SD)	78 (64)	87 (80)	< 0.01

*Data from Web-based Analysis of Dialysis Data Archives system by Japanese Society for Dialysis Therapy, ver. 2.1. Values are shown as *n* (%); SD, standard deviation.

A total of 357 facilities (8% of surveyed facilities, 22% of responding facilities, and 65% of facilities providing exercise instruction) had already claimed the fees for exercise instruction during haemodialysis treatment. Insurance claims had been made by 66% of the clinics and 65% of the hospitals among the 545 facilities. The facilities claiming fees had more haemodialysis beds and patients on haemodialysis than those that had not claimed (Table 3).

**Table 3:** Factors affecting claims for fees for exercise instruction during haemodialysis treatment.

	Facilities that have claimed (n = 357)	Facilities that have not claimed (n = 188)	P value
Type of facility, n (%)			
Clinic	185 (66%)	94 (34%)	0.72
Hospital	170 (65%)	93 (35%)	
Number of inpatient beds, n (%)			
0	91 (59%)	62 (41%)	0.39
1–19	35 (66%)	18 (34%)	
20–199	113 (68%)	52 (32%)	
> 200	79 (63%)	47 (37%)	
Number of inpatient beds, median (IQR)	50 (0, 199)	44 (0, 216)	0.51
Number of haemodialysis beds, n (%)			
< 19	43 (57%)	32 (43%)	0.18
20–39	144 (63%)	83 (37%)	
40–59	91 (69%)	40 (31%)	
> 60	73 (71%)	30 (29%)	
Number of haemodialysis beds, median (IQR)	36 (25, 55)	31 (22, 50)	0.03*
Number of patients on haemodialysis, n (%)			
< 50	88 (64%)	50 (36%)	0.24
51–100	124 (62%)	75 (38%)	
> 101	138 (70%)	59 (30%)	
Number of patients on haemodialysis, median (IQR)	89 (50, 150)	78 (49, 123)	0.02*

Values are shown as *n* (%), or median (IQR).

IQR, interquartile range, *p < 0.05.

Of the 550 facilities, 245 (55%) started providing exercise instruction in or after April 2022, the date from when insurance claims could be made. In facilities that started this provision after claims approval, 91% had actually claimed. However, only 45% of facilities that had provided this instruction before the change in the insurance regime had already claimed at the time of the survey.

### Details of exercise instruction

In total, 85% of the 550 facilities providing exercise instruction used the JSRR clinical practice guideline for renal rehabilitation. Exercise therapy was provided to a median (IQR) of 8.5 (4, 17) patients, a median (IQR) percentage of 11% (4%, 26%) of the total patients on haemodialysis at the facility. Overall, 58% of facilities provided exercise therapy to 10 or fewer patients. Making a claim was not associated with the number of patients given instruction (claimed: 8 (4, 17), not claimed: 9 (4, 16), p = 0.96). Facilities with PTs tended to provide exercise instruction to more patients than those without PTs (with PTs: 10 (4, 20), without PTs: 7 (4, 15), p = 0.06).

**Figure 1:**
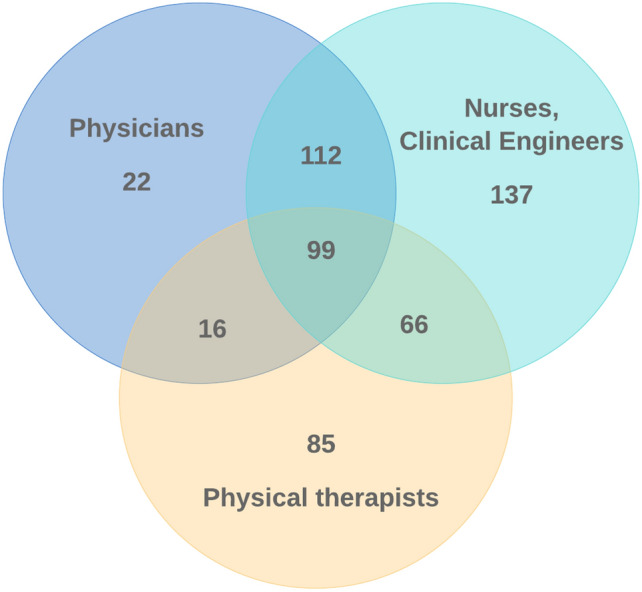
Venn diagram of instructors providing exercise instruction during haemodialysis treatment. “Physical therapists” includes physical therapists, occupational therapists, and health fitness programmers.

The details of the instructors are shown in Table 4 and Fig. 1. Multiple responses were possible, but the instructors included physicians in 45% of facilities, nurses in 74%, and clinical engineers in 24%. In 266 facilities (48%), instruction was provided by PTs either alone or with someone else. Within the PTs category, the provision was by PTs at 253 (46%) facilities, OTs at 49 (9%), and health fitness programmers at five (0.1%). Some facilities had instruction provided by several other professions, including nutritionist, clinical laboratory technician, and radiological technologist, but these did not fall within the claims requirements. There were differences in claim rates by type of instructor, with higher claim rates for physicians (81%) and PTs (71%) than for nurses (67%) and clinical engineers (48%). Of the 25% of facilities where only nurses or clinical engineers were instructors, only 45% had claimed the fee, much fewer than facilities using other types of instructors.

**Table 4:** Details of instructors used by facilities.

Type of instructors (multiple answers possible)	Facilities	Percentage that had claimed the fee
Physicians + PTs + nurses/clinical engineers	99	84%
Physicians + PTs	16	94%
Nurses/clinical engineers + PTs	66	62%
PTs only	85	60%
Physicians + nurses/clinical engineers	112	78%
Physicians only	22	73%
Nurses/clinical engineers only	137	45%
Total	550	65%
P value		< 0.01

Values are shown as *n,* % or median (interquartile range).

PTs include physical therapists, occupational therapists, and health fitness programmers.

*p < 0.05.

Overall, 537 (98%) of the facilities provided exercise instruction during haemodialysis (Table 5), and 23% of the facilities also provided it before/after haemodialysis or on non-haemodialysis days. The frequency of the instruction was three times a week for 80%, twice a week for 9%, and once a week or less for 11% (Supplementary Table S3). The length of the instruction session was less than 20 min for 19%, between 20 and 30 min for 66%, and more than 30 min for 15%.

**Table 5:** Timing of exercise instruction during haemodialysis treatment.

	Facilities
During or around haemodialysis treatment	537 (98%)
During haemodialysis only	412 (75%)
During haemodialysis + before or after haemodialysis	60 (11%)
During haemodialysis + non-dialysis day	22 (4%)
During/before or after haemodialysis + non-dialysis day	43 (8%)
Before or after haemodialysis only	0
Non-dialysis day only	1 (0%)
No exercise therapy	6 (1%)
N/A	6 (1%)

Values are shown as* n* (%).

**Figure 2:**
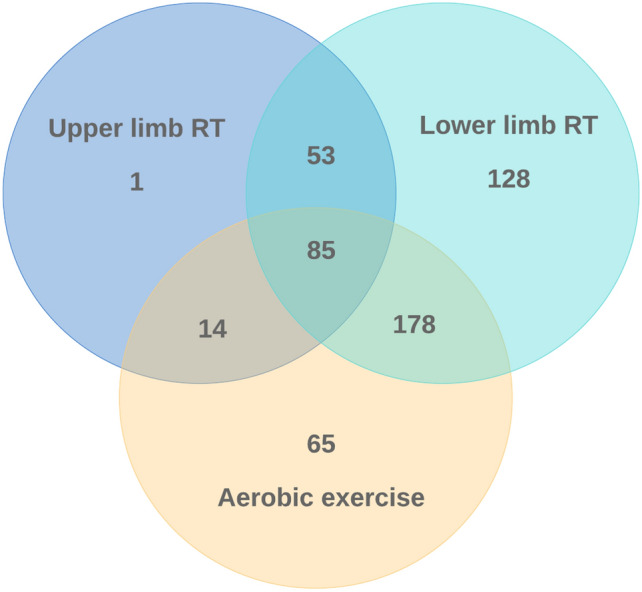
Venn diagram of types of exercise instruction provided during haemodialysis treatment. RT, resistance training.

The details of the types of exercise instruction provided are shown in Table 6 and Fig. 2. The most frequently performed item was lower limb resistance training (81%), followed by aerobic exercise (62%, multiple answers possible). Nutritional instruction was provided at 24% of the facilities. The most common combination was training combining aerobic exercise and lower limb resistance training (48%). There were no differences in provision of exercise instruction by type of facility or presence of PTs.

**Table 6:** Types of exercise instruction during haemodialysis treatment.

Type (multiple answers possible)	Total	Type of facility		Physical therapists	
		Hospital	Clinic	Presence	Absence
Aerobic exercise	342 (62%)	169 (64%)	173 (62%)	185 (34%)	157 (29%)
Upper limb resistance training	153 (28%)	79 (30%)	74 (26%)	95 (17%)	58 (11%)
Lower limb resistance training	444 (81%)	224 (84%)	220 (78%)	242 (44%)	202 (37%)
Balance exercise	50 (9%)	24 (9%)	26 (9%)	27 (5%)	23 (4%)
Group exercise	33 (6%)	16 (6%)	17 (6%)	16 (3%)	17 (3%)
Nutrition instruction	131 (24%)	57 (21%)	74 (26%)	66 (12%)	65 (12%)
Others	54 (10%)	29 (11%)	25 (9%)	26 (5%)	28 (5%)
P values		0.83		0.44	

Values are shown as *n* (%). *p < 0.05.

**Figure 3:**
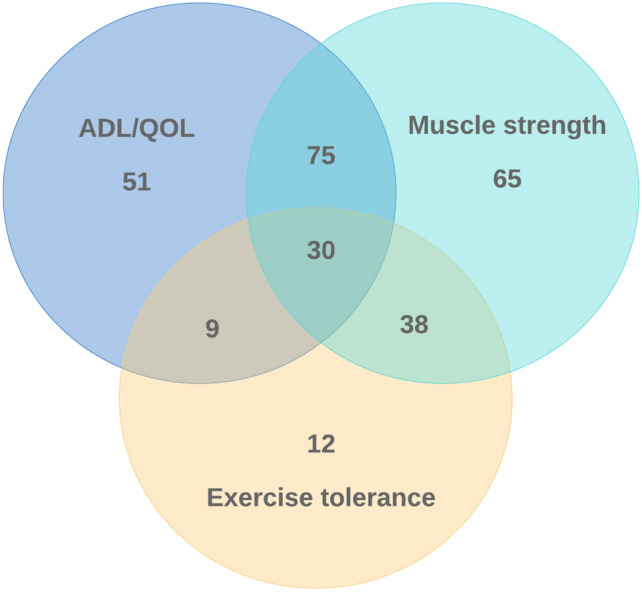
Venn diagram of items used to evaluate exercise instruction. ADL/QOL, activities of daily living and/or quality of life.

Evaluation of the effectiveness of the instruction was not a requirement for claims, but it was performed in 76% of the facilities (Fig. 3, Table 7). The most frequently evaluated item was change in muscle strength (49%), followed by change in ability to carry out activities of daily living and quality of life (ADL/QOL, 39%, multiple answers). Exercise tolerance was evaluated in 89 (21%) facilities, and included cardiopulmonary exercise testing (6%) and the 6-min walk test or incremental shuttle walk test (18%). Overall, 76% of the facilities evaluated either exercise tolerance or muscle strength. The evaluators tended to be PTs in hospitals and physicians and nurses in clinics (Supplementary Table S4). Clinical engineers provided evaluations in 17% of the facilities, mainly clinics. A total of 81% of facilities responded that they would continue exercise therapy after the 90-day claimable period had expired, and 84% responded that the exercise instruction was effective.

**Table 7:** Items used to evaluate exercise instruction.

Evaluation items (multiple answers)	n (%)
Any evaluation	403 (73%)
Exercise tolerance (cardiopulmonary exercise test)	33 (6%)
Exercise tolerance (e.g. 6-min walk test or incremental shuttle walking test)	99 (18%)
Short physical performance battery	61 (11%)
Muscle strength	268 (49%)
Nutrition status	149 (27%)
ADL/QOL	212 (39%)
No evaluation	147 (27%)

ADL/QOL, activities of daily living and quality of life.

Values are shown as *n* (%).

### Adverse events associated with exercise therapy during haemodialysis

Overall, 39% (214) of the facilities had experienced adverse events associated with exercise instruction during haemodialysis. Most were minor and did not require any specific treatment (Table 8). However, 18 facilities reported moderate-to-severe adverse events, and a secondary survey was conducted to obtain more information. Eighteen adverse events were reported from 11 hospitals and seven clinics. They included 12 cases of cardiovascular problems (11 arrhythmias and one case of chest discomfort) and six cases of haemodialysis circuit problems. The period from the start of exercise instruction to the onset of the adverse events was 1 day for three cases, 2–28 days for six cases, 29–90 days for five cases, and 91 days or more for four cases. The type of exercise being performed at the time of the occurrence of the adverse event was lower limb resistance training in eight cases, aerobic training in 11 cases, and group exercises in one case. The 11 arrhythmias were all sinus tachycardia. One of the six cases of haemodialysis circuit problems required re-puncture because the haemodialysis needle had almost been removed. The remaining five cases showed increased venous pressure caused by flexion of the arm with arteriovenous fistula, which was improved by arm repositioning. None of the 18 people required hospitalization and there were no ongoing effects. In two cases, exercise instruction was discontinued after the event, but the others continued.

**Table 8:** Adverse events associated with exercise therapy during haemodialysis treatment.

Adverse events (multiple answers)	n (total 550 facilities)
Moderate–severe adverse events	18 facilities
Chest discomfort	1
Arrhythmia/abnormal heartbeat	12
Haemodialysis circuit problems	6
Falls/trauma	0
Haemodialysis needle removal	0
Angina/myocardial infarction	0
Cerebrovascular disorders	0
Minor adverse events	214 facilities
Decreased blood pressure	106
Increased blood pressure	57
Muscle/joint pain	73
Feeling unwell/dizziness	33
Skin problems	8
Other	11

Values are shown as n.

### Other findings

Of the 1103 facilities that did not provide exercise instruction during haemodialysis treatment as of March 2023, 44% planned to start in the future. The main reasons for not providing exercise instruction were staff shortages (64%) and being unable to meet the requirements for claims (36%, Table 9).

**Table 9:** Reasons for not providing exercise instruction during haemodialysis treatment.

Reasons (multiple answers possible)	n = 1103
Staff shortages	709 (64%)
Unable to meet claim requirements	395 (36%)
Equipment shortage	290 (26%)
Insufficient knowledge	224 (20%)
No patient request	158 (14%)
Lack of interest	20 (2%)
Other	208 (19%)

Values are shown as n (%).

Exercise therapy was provided for patients with non-dialysis CKD at 154 facilities (9% of responding facilities and 4% of those sent the survey). Of these, 118 (76%) were hospitals, and 59% had provided instruction to fewer than 10 people. The reasons for not providing exercise instruction were staff shortages (49%), because the additional fee cannot be claimed for non-dialysis CKD patients (25%), and absence of appropriate patients (22%) (Supplementary Table S5).

## Discussion

In this nationwide survey, we evaluated the situation following the approval of insurance claims for exercise therapy during haemodialysis treatment. To our knowledge, Japan is the first country in the world to approve this treatment for insurance payouts, and it may therefore be a reference for countries and regions that consider adding this coverage in the future. At least 13% of haemodialysis facilities in Japan provided exercise instruction during haemodialysis treatment, and 65% of these facilities claimed the additional fee. Exercise instruction was generally lower limb resistance training and aerobic exercise or a combination of these, with 66% of the facilities focusing on muscle strength to determine effectiveness. No life-threatening adverse events were reported.

Our survey was conducted relatively soon (11 months) after the addition of this new fee, but the number of facilities providing exercise instruction had nearly doubled (298–550) in the period, confirming the increased popularity of this approach after claims approval. Reasons for not providing exercise instruction were mainly staff availability and inability to meet claims requirements. However, 35% of the facilities provided exercise instruction despite being unable to claim. Several facilities planned to begin providing exercise instruction in the future, and further growth is expected. Our results suggested that the initiation of exercise instruction in haemodialysis facilities requires the establishment of instructional content and evaluation items, as well as the recruitment of instructors and evaluators. Training by clinical engineers alone was not eligible for a claim, but clinics often had non-PT medical staff serving as instructors.

It is difficult to use our results to discuss whether the claim requirements are appropriate. Previous meta-analyses have reported that structured exercise instruction for patients on haemodialysis was effective in reducing depressive symptoms and increasing functional capacity^4^. However, the effects on mortality and cardiovascular events were unclear^4^. Exercise therapy during haemodialysis treatment included in this meta-analysis consisted mainly of aerobic and resistance training for at least 20 min three times a week^4^. Our survey showed the majority of facilities provided the minimum level required to claim (20 min). A previous study also found that exercise therapy was more effective in improving exercise tolerance if continued for more than 6 months^13^. Further evaluation is needed to determine whether there is prognostic benefit to the practice as it is performed in the real world, including whether the limited 90-day claims period is appropriate.

A recent meta-analysis comparing the effects of nine different exercise modalities reported that aerobic exercise alone or aerobic exercise plus resistance training contributed most strongly to improvements in exercise tolerance^14^. The JSRR clinical practice guidelines recommend both aerobic exercise and resistance training, but many facilities focused on lower limb resistance training, which is easier to perform. Aerobic exercise requires equipment such as ergometers, which may be one reason why it was used less often. We also concluded that the guidelines have been popular and frequently used by providers.

The evaluation of exercise instruction was not essential to make a claim but was performed at 76% of the facilities. The evaluation items were mainly changes in muscle strength and ADL/QOL, which can easily be measured. Few facilities evaluated exercise tolerance. The JSRR clinical practice guidelines recommend using muscle strength and exercise tolerance as evaluation items^5^. Exercise tolerance is necessary for accurate evaluation of the effects of exercise instruction.

This was a facility-based cross-sectional survey, and we therefore could not evaluate individual patients, including their baseline ability to perform activities of daily living, comorbidities, nutrition status, exercise regimes, and patient outcomes other than adverse events. However, therapeutic nutrition instruction combined with exercise therapy has been reported to affect outcomes, including muscle strength, in older people^15^ and patients on dialysis^16^. The JSRR plans to start a registry cohort study and carry out a prospective evaluation to determine the effect of the social strategy of insurance claims approval for exercise instruction on patient outcomes.

Our results are consistent with previous reports that adverse events due to exercise therapy during haemodialysis treatment are often minor^17^. The nature of a questionnaire-based survey means that we cannot accurately determine that all adverse events were included, and therefore cannot completely guarantee the safety of exercise therapy during haemodialysis treatment based on our analysis. However, the novelty of this study is that no severe adverse events were reported in a real-world survey. Safety evaluation, including the need for cardiovascular screening and prevention of needle removal, should continue.

Provision of exercise therapy among people with non-dialysis CKD was included in the survey, but this involved very small numbers. This may be because insurance claims for exercise instruction for patients with non-dialysis CKD have not been approved in Japan, and also because the survey was conducted in haemodialysis facilities. The usefulness of exercise therapy for patients with non-dialysis CKD is gradually being established^18,19^. In the longer term, we hope that claims will also be approved for exercise therapy for patients with non-dialysis CKD.

This study had several limitations. First, the survey was sent to almost all the haemodialysis facilities in Japan, but fewer than half responded. Our response rate of 38.9% of surveyed facilities is comparable to 67% and 29% in previous studies^9,10^. Response bias was also possible because it is likely that more responses were received from facilities that provided exercise instruction than those that did not. We therefore think that it is more accurate to compare the provision rate by providing/surveyed (13%) than by providing/responding (33%). A comparison of the characteristics of responding and surveyed facilities indicated that the differences between the two groups were not clinically significant and that the responding facilities were representative of the surveyed facilities. Second, the nature of the questionnaire-based survey meant that we were unable to confirm the results with receipt data. Recall bias may also have affected data on start dates. Third, this was a cross-sectional study, and we could only assess the situation regarding exercise instruction at each facility. We could not assess patient characteristics, details of the type of exercise, the effectiveness of the exercise and/or nutritional instruction, and the prognostic impact of instructor type. A prospective registry analysis will be conducted in the future to evaluate the prognostic impact of exercise instruction within the criteria for insurance claims.

In conclusion, because it has been possible to claim for fees for exercise instruction during haemodialysis treatment, this therapy has become more popular, and more patients on haemodialysis are now undergoing exercise therapy. Future studies will evaluate the prognostic impact of fees on exercise instruction during haemodialysis treatment in a nationwide prospective study.

### Supplementary Information

Supplementary Tables.

## Data Availability

The data underlying this article will be shared upon reasonable request to the corresponding author.
