# Supplementary material for: Exercise instruction during haemodialysis treatment after changes to the insurance regime: a nationwide questionnaire survey in Japan
Source: Sci Rep. 2024 Apr 22;14:9171. doi: 10.1038/s41598-024-59995-4 (PMC11035573; doi:10.1038/s41598-024-59995-4)
Supplement: Supplementary file 1 — Supplementary Tables. [file 41598_2024_59995_MOESM1_ESM.pdf]

Supplementary information

Exercise instruction during haemodialysis treatment after changes to the insurance regime: a nationwide questionnaire survey in Japan.

Tadashi Sofue[1], Ryota Matsuzawa[2], Hiroki Nishiwaki[3], Yohei Tsuchida[4], Keisei Kosaki[5], Junichi Hoshino[6], Ichiei Narita[7], Kunihiro Yamagata[8]

1 Department of Cardiorenal and Cerebrovascular Medicine, Kagawa University, Kagawa, Japan

2 Department of Physical Therapy, School of Rehabilitation, Hyogo University of Health Sciences, Kobe, Japan

3 Division of Nephrology, Department of Internal Medicine Showa University Fujigaoka Hospital, Yokohama, Japan

4 Department of Nephrology, Shinrakuen Hospital, 3-3-11 Shindori-Minami, Nishi-Ku, Niigata, Japan

5 Institute of Health and Sport Sciences, University of Tsukuba, 1-1-1 Tennodai, Tsukuba, Ibaraki, Japan

6 Department of Nephrology, Tokyo Women's Medical University, Tokyo, Japan

7 Division of Clinical Nephrology and Rheumatology, Niigata University Graduate School of Medical and Dental Sciences, Niigata, Japan

8 Department of Nephrology, Faculty of Medicine, University of Tsukuba, Tsukuba, Ibaraki, Japan

Supplementary Table S1. Claims requirements for exercise instruction during haemodialysis treatment

	Claims requirements
Patients	Patients on haemodialysis
Instruction provided	Exercise therapy or other instruction
Instructor	Doctor, PT, or OT who has received specific instruction on exercise or nurse who has received specific direction from a doctor
Maximum patients per instructor	20 patients for doctors, PTs and OTs, and 8 patients for nurses
Implementation time	More than 20 consecutive minutes during a single haemodialysis treatment
Limited duration	90 days from the date of initiation
Additional fee	75 extra points (750 yen)
Notes	Refer to the clinical practice guidelines for renal rehabilitation

PT, physical therapist; OT, occupational therapist

Supplementary Table S2. Factors affecting the performance of exercise instruction during haemodialysis treatment

		Facilities with instruction (n = 550)	Facilities without instruction (n = 1021)	P value
Type of facility, n (%)	Clinic	281 (35%)	513 (65%)	0.13
	Hospital	266 (31%)	572 (69%)	
Number of inpatient beds, n (%)	0	153 (34%)	292 (66%)	0.20
	1–19	54 (36%)	94 (64%)	
	20–199	165 (37%)	285 (63%)	
	> 200	129 (30%)	299 (70%)	
Number of inpatient beds, median (IQR)		50 (0, 205)	90 (0, 282)	0.03*
Number of haemodialysis beds, n (%)	< 19	76 (20%)	302 (80%)	< 0.01*
	20–39	230 (34%)	443 (66%)	
	40–59	132 (36%)	235 (64%)	
	> 60	103 (51%)	99 (49%)	
Number of haemodialysis beds, median (IQR)		34 (24, 52)	28 (18, 41)	< 0.01*
Number of patients on haemodialysis, n (%)	< 50	140 (25%)	427 (75%)	< 0.01*
	51–100	201 (35%)	373 (65%)	
	> 101	198 (43%)	267 (57%)	
Number of patients on haemodialysis, median (IQR)		84 (50, 135)	63 (34, 101)	< 0.01*

Values are shown as *n* (%), or median (IQR)

IQR, interquartile range. * $p < 0.05$

Supplementary Table S3. Frequency and length of exercise instruction during haemodialysis treatment

	n (%)
Frequency	
Once a week or less	59 (11%)
Twice a week	47 (9%)
Three times a week	435 (80%)
Length	
Less than 20 minutes	104 (19%)
20–30 minutes	358 (66%)
More than 30 minutes	80 (15%)

Values are shown as *n* (%)

Supplementary Table S4. Evaluators of exercise instruction during haemodialysis treatment

Types of evaluators (Multiple answers)	Total (n = 417)	Hospital (n = 216)	Clinic (n = 201)
Physicians	118 (28%)	44 (20%)	73 (36%)
Nurses	200 (48%)	86 (40%)	114 (57%)
Clinical engineers	72 (17%)	23 (11%)	49 (24%)
Physical therapists	221 (53%)	160 (74%)	76 (38%)
P value		< 0.01*	

Values are shown as *n* (%), * $p < 0.05$

Physical therapists include physical therapists, occupational therapists, and health fitness programmers

Supplementary Table S5. Reasons for not providing exercise instruction for patients with non-dialysis CKD.

Reasons (multiple answers possible)	n = 1503
Staff shortages	742 (49%)
Because the additional fee cannot be claimed for non-dialysis CKD patients	378 (25%)
Equipment shortage	208 (14%)
Insufficient knowledge	262 (17%)
No appropriate patients	323 (22%)
Lack of interest	32 (2%)
Other	319 (21%)

Values are shown as n (%).
